# Combination antibiotic therapy is required to eliminate *Bartonella henselae* in multiple microenvironments

**DOI:** 10.3389/fmicb.2026.1726180

**Published:** 2026-04-02

**Authors:** Emily L. Olsen, Monica E. Embers

**Affiliations:** Division of Immunology, Tulane National Biomedical Research Center, Tulane University School of Medicine, Covington, LA, United States

**Keywords:** antibiotic, azlocillin, *B. henselae*, *Bartonella*, biofilm, DH82 cells, gentamicin, rifampin

## Abstract

*Bartonella* are gram-negative, facultative intracellular bacteria. Infection by *Bartonella* manifests as different clinical syndromes collectively known as bartonellosis. The well-known diseases caused by these bacteria are cat scratch disease (*Bartonella henselae*), trench fever (*Bartonella quintana*) and Carrion’s disease (*Bartonella bacilliformis*). Excluding *B. bacilliformis*, which is evolutionarily more distinct than the other species, *Bartonella* infections result in self-limiting disease that is often undiagnosed and untreated. However, individuals with compromised immune systems or other undefined conditions may experience clinical manifestations, which can become life-threatening and need to be treated with effective antibiotics. To date, there is no standard treatment course for these infections, and many doctors prescribe antibiotics based on limited case studies. Within the host, *Bartonella* can grow extracellularly, intracellularly, and in biofilms. To determine an effective antibiotic strategy, it is important to understand *Bartonella* susceptibility in each of these growth conditions. We hypothesized that combination antibiotic treatments are required to effectively eliminate *Bartonella henselae* growth, particularly in biofilm and intracellular environments. Our previous work has shown that *B. henselae* treatment with single antibiotics in different media, as well as in DH82 canine macrophages, was ineffective in eliminating bacteria. We expanded this work with different antibiotics supported by case reports, as well as double and triple combinations. The following antibiotics were tested: doxycycline, gentamicin, azithromycin, azlocillin, rifampin, and clarithromycin. We found that while monotherapy may inhibit growth extracellularly, it is ineffective when used against intracellular bacteria or pre-existing biofilms. Gentamicin in combination with either rifampin or azlocillin significantly reduced bacterial growth in multiple microenvironments. The effectiveness of combination therapy supports the notion that *Bartonella* species utilize host cells and biofilms as antibiotic evasion strategies.

## 1 Introduction

Bacteria within the genus *Bartonella* are gram-negative coccobacilli in the class proteobacteria, with over 35 named species, of which at least seven have been described in human cases (Breitschwerdt, 2017; Tahmasebi Ashtiani et al., 2024). *Bartonella* spp. are facultative intracellular bacteria, with target cells including, but not limited to, endothelial cells, red blood cells, and macrophages. Other cell types have been infected *in vitro*, suggesting that there are more niches in the host for this pathogen that remain to be elucidated. Additionally, *Bartonella* spp. have been identified as biofilms in human hosts, namely around the heart in cases of blood culture-negative endocarditis (BCNE) (Meidrops et al., 2023; Xi et al., 2024). The three most prominent species which cause human disease are *B. henselae* (causative agent of cat-scratch disease), *B. quintana* (causative agent of trench fever), and B. bacilliformis (causative agent of Carrion’s disease). However, numerous case reports demonstrate that a variety of *Bartonella* species can also cause clinical disease in humans (Delaney et al., 2024).

Bartonellosis, the term used to collectively describe *Bartonella* spp. infections, encompasses a range of clinical signs and symptoms that vary from mild manifestations such as fever and malaise to more serious complications like endocarditis, bacillary angiomatosis, and various neurological manifestations. Bartonellosis is extremely difficult to diagnose clinically due to the broad disease presentation as well as the need for highly sensitive diagnostic tools. Current methods of detecting *Bartonella* spp. include serological testing, blood cultures, and PCR. Clinical diagnosis is weighted heavily with exposure to felines (CDC, 2023) or known risk factors such as infestation with body lice. In many patients, *Bartonella* spp. infection is mild and may not require antibiotic treatment. However, individuals with high risk factors for severe disease, such as prior tissue damage or a compromised immune system, can progress to more serious forms of the disease which require antibiotic therapy.

### 1.1 Treatment for infection

Current guidelines for the treatment of bartonellosis are based on disease presentations (Angelakis and Raoult, 2014) and patient-specific contraindications. For example, patients with prior renal impairments may be administered an alternative to gentamicin, which is known to cause kidney damage. Physicians and veterinarians thus rely heavily on case reports to determine the best course of treatment for their patients. Studies demonstrating antibiotic efficacy are limited to *in vitro* experiments, small clinical trials, or limited meta-analyses (Prutsky et al., 2013). Treatment failure has been documented in as high as 39% of patients (Pizzuti et al., 2024), with the majority experiencing adverse events, acute toxicity to the drugs, or the need to escalate therapy. A list of the antibiotics used in these experiments and their characteristics are summarized in Table 1.

**TABLE 1:** Antibiotics that were used for *in vitro* efficacy testing.

Antibiotic	Class	Mechanism of action	Bactericidal/bacteriostatic
Doxycycline (Parmar and Patel, 2025)	Tetracycline	Binds 30S ribosomal subunit, preventing protein synthesis and growth	Bacteriostatic
Gentamicin (Chaves and Tadi, 2023)	Aminoglycoside	Inhibits ribosome functionality, disrupting protein synthesis	Bactericidal
Azithromycin (Patel and Hashmi, 2025)	Macrolide	Inhibits 50S ribosomal subunit, preventing protein synthesis and growth	Bacteriostatic
Azlocillin (Sanders, 1983)	β-Lactam	Prevent synthesis of bacterial cell wall by binding to PBP during peptidoglycan cross-linking	Bactericidal
Rifampin (Patel and Alan, 2023)	Ansamycin	Inhibits DNA-dependent RNA polymerase, blocking RNA synthesis	Bactericidal
Clarithromycin (Patel and Hashmi, 2025)	Macrolide	Inhibits 50S ribosomal subunit, preventing protein synthesis and growth	Bacteriostatic

Treatment for bartonellosis is highly dependent on disease presentation and *Bartonella* species (Mikes et al., 2025). Treatment options can vary from no antibiotics in mild cat scratch disease to combination therapy delivered via intravenous infusions in complicated presentations such as nervous system involvement. One of the main antibiotics used for multiple presentations of bartonellosis is doxycycline (Prutsky et al., 2013), a semi-synthetic derivative in the tetracycline class with a broad range of antimicrobial activity. This antibiotic is often prescribed for other skin infections as well as in Lyme disease, caused by *Borrelia burgdorferi*, of which *Bartonella* spp. can be present as a co-infection (Parmar and Patel, 2025). Like other tetracyclines, doxycycline is a bacteriostatic antibiotic and confers high resistant rates in many species of bacteria (Rissardo and Caprara, 2019) although the exact mechanisms of *Bartonella* spp. resistance are not well characterized. It is thought that *Bartonella* spp. utilize their ability to enter host cells as a method of intrinsic antibiotic resistance rather than active resistance conferred by plasmids (Biswas and Rolain, 2010; Xi et al., 2024).

Other antibiotics are usually prescribed as part of a combination therapy cocktail during complicated bartonellosis, which can include but are not limited to gentamicin (Mikes et al., 2025), azithromycin (Ives et al., 2000; Margileth, 1992), clarithromycin (Ives et al., 2000), and rifampin (Margileth, 1992). Rifampin was found to have some activity against *B. henselae* biofilms (Zheng et al., 2020) after 6 days of treatment but was ultimately more effective when used in combination with other antibiotics. Rifampin was also shown to have activity against intra-erythrocytic *B. quintana*, although this activity was much less effective compared to the aminoglycoside gentamycin (Rolain et al., 2003) Azlocillin, also included in this study, has recently been shown to have antimicrobial activity against both *Borrelia burgdorferi* (Pothineni et al., 2020) and *Bartonella henselae* (Gadila and Embers, 2021), making it an attractive potential candidate for treatment in these co-infected patients. However, it is important to consider the relative toxicities to antibiotics when longer courses of antimicrobial cocktails are prescribed, which limits doctors’ options for extended therapy during chronic *Bartonella* spp. infections. A full table with each antibiotic and its concentrations, typical human doses, and the maximum serum concentrations (if reported) are listed in Table 2.

**TABLE 2:** Antibiotic concentrations used in experiments.

Antibiotic	Concentrations* tested for MIC/Singular MBC (μg/mL)	Human adult equivalent dose	Example C_max_ in adults (μg/mL)
Doxycycline	10, 5, 2, 1, 0.5, 0.3, 0.1, 0.01, 0.001	100–300 mg given orally per day (Parmar and Patel, 2025)	2.6 μg/mL after 2 h of 200 mg dose (National Academies of Sciences Engineering and Medicine, 2020)
Gentamicin	16, 8, 4, 2, 1, 0.5, 0.3, 0.1, 0.01	5–7 mg/kg given either intravenously or intramuscularly per day for mild to systemic infections (Chaves and Tadi, 2023)	Average 19 μg/mL after similar dose given (Allou et al., 2016)
Azithromycin	10, 5, 2, 1, 0.5, 0.3, 0.1, 0.05, 0.01, 0.005, 0.001	250–1,000 mg given orally or intravenously, depending on severity of infection (Sandman and Iqbal, 2024)	0.5–2.5μg/ml after 20 mg/kg dose (Bandyopadhyay et al., 2025)
Azlocillin	5, 2, 1, 0.5, 0.3, 0.1, 0.05, 0.01, 0.005, 0.001	6–15 g given intravenously per day, depending on severity of infection (Neu et al., 1983; Parry, 1983)	Up to 500 μg/mL right after injection (Singlas and Haegel, 1984)
Rifampin	10, 5, 2, 1, 0.5, 0.3, 0.1, 0.05, 0.01, 0.005, 0.001	Up to 600 mg given intravenously or orally per day (Patel and Alan, 2023)	8.2 μg/mL average after 600 mg dose (Abulfathi et al., 2019)
Clarithromycin	10, 5, 2, 1, 0.5, 0.3, 0.1, 0.05, 0.01, 0.005, 0.001	125–500 mg given orally per day (Patel and Hashmi, 2025)	1.01–2.85 μg/mL, depending on oral dose (Rodvold, 1999)

*Concentrations were tested in a 1:1 ratio.

In addition to antimicrobials, additional treatments may be prescribed to patients with immune-mediated pathologies. The most common of these are steroids; however, this is controversial as they are also used to suppress the immune system, which in turn increases the opportunity for further *Bartonella* colonization in the host. Oral steroids with a tapering dose are often given to patients presenting with neuroretinitis. Another promising therapy, particularly with conditions related to immunosuppression or immune-mediated pathologies, is intravenous immunoglobulin infusions (IVIG) (Dietmann et al., 2022; Rissardo and Caprara, 2019). This treatment has been documented in several case reports of children infected with *Bartonella* that have developed neurological complications secondary to bartonellosis.

### 1.2 Importance

The goal of this study was to systematically measure the efficacy of candidate antibiotics against *B. henselae* when grown extracellularly, intracellularly, and as a biofilm. While many studies examine antibiotic efficacy through minimum inhibitory concentrations (MICs) and minimum bactericidal concentrations (MBCs), we used both measurements as well as bacterial killing in different growth conditions that better model the different microenvironments this pathogen can occupy in the human host. We hypothesized that combinations of antibiotics are required to fully eliminate Bartonella growth in these different growth conditions.

## 2 Materials and methods

### 2.1 Bartonella culturing

*Bartonella henselae* strain San Antonio 2 was used in these experiments. Bacterial stocks were grown in both Schneider’s Drosophila Medium (Thermofisher, Cat No. 21720024) and Grace’s Insect Medium (Gibco, 11605–094) and frozen in media supplemented with 50% glycerol. For use in assays, a frozen stock of *B. henselae* was streaked onto blood agar plates (Remel, R01198) and incubated at 37°C with 5% CO_2_ for 7–10 days. After incubation, isolated colonies were used to inoculate either Grace’s media or Schneider’s media. After 5–8 days in growth media, cultures were harvested. The culture was diluted to an OD_600_ of 0.1 using the SmartSpec 300 (Bio-Rad, 170–2501) before use in assays, for an approximate count of 3 × 10^7^ colony-forming units (CFU)/mL.

### 2.2 Preparation of antibiotics

The following antibiotics were used in these assays: doxycycline (Sigma, D9891-1G) gentamicin (FisherSci, BP918-1) azithromycin (TCI, 117772-70-0), azlocillin (provided by Flightpath Biosciences, Inc.), rifampin (Acros, 45562), and clarithromycin (Acros, 45522). Antibiotic stocks were created by dissolving singular antibiotics in 100% molecular-grade ethanol (rifampin) at 1 mg/ml or sterile cell-culture grade water (all others). Stock solutions were stored at −20°C until use. All antibiotics were diluted fresh in the respective media on the day of their use in the assays.

### 2.3 Extracellular antibiotic testing

On the day of the assay, *Bartonella* species were diluted in their respective medias as previously described to an OD of 0.1. In each well of a 96 well plate, 50 μL of OD 0.1 bacteria was added to 50 μL of media with the respective dilution of antibiotics for a total approximate concentration of 1.5 × 10^7^ CFU/mL. Table 2 shows a full list of dilutions for each antibiotic. Antibiotic concentrations used in the experiments are listed on the center column and typical adult human doses for mild or systemic infections are given on the right column. The duration of antibiotic administration in humans is highly dependent on infection type and severity. For complicated Bartonella infections, multiple antibiotics can be prescribed up to months at a time (Biswas and Rolain, 2010; Prutsky et al., 2013; Raoult et al., 2003). Wells containing only bacteria or only media served as the positive and negative controls, respectively. Plates were incubated at 37°C in 5% CO_2_ for 4 days. After incubation, the OD_600_ of each plate was read using the SmartSpec 300 (Bio-Rad, 170–2501). The MIC was determined by comparing the control wells to experimental conditions using a cut-off with the negative control for growth. This assay was repeated three times with all six antibiotics.

**FIGURE 1:**
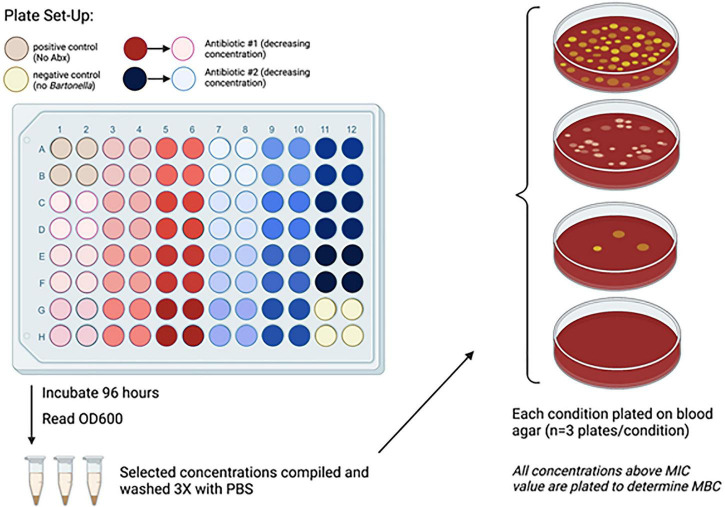
Experimental schematic depicting the minimum inhibitory/bactericidal concentration (MIC/MBC) assay for extracellular *Bartonella*. *B. henselae* grown in either Grace’s or Schneider’s Media was diluted to an OD_600_ of 0.1 for the extracellular MIC/MBC assays. Antibiotics diluted in the same media were added in a 1:1 ratio with bacterial slurry. After 96 h of incubation, inhibition of growth (MIC) was determined by comparing absorbence values between respective antibiotic concentrations with control wells containing only media and wells with only bacteria. Wells containing antibiotics that successfully inhibited bacterial growth were plated on a single blood agar plate to determine bactericidal activity (MBC) and were reported as either positive or negative for growth. Combination therapies were tested for bactericidal activity only, and bacteria remaining after 96 h of antibiotic exposure were plated in triplicate for quantification. For all assays, the wells for each respective condition were combined and antibiotics were removed from solution by washing with PBS three times before plating. Only the control wells containing *B. henselae* without antibiotics were serial diluted for enumeration. Colonies were counted after 14 days of incubation at 37°C in 5% CO_2._ Significant reduction of bacterial counts (CFU) from combination therapy was determined as the difference between the antibiotic-free control and the antibiotic-treated bacterial count at each specified concentration.

Contents of the wells from the MIC assays that showed no bacterial growth were used for the MBC assay. To collect all remaining bacteria possible, these wells were vigorously washed and scraped with a pipette tip, and the contents of all replicates were collected into 1.5 mL tubes. Tubes were centrifuged at 6,000 × g for 10 min. The supernatant was removed, and the pellet was resuspended with 500 μL of 1X PBS. This was repeated for a total of 2 washes. After the second wash, the pellet was then resuspended with 100 μL of PBS and plated onto blood agar plates. The pellets from the control tubes were resuspended with 500 μL of PBS and serially diluted to determine quantification of bacterial load. The plates were examined for bacterial growth after incubating for 14 days at 37°C in 5% CO_2._ This assay was repeated 3 times with each antibiotic. The methods for these assays are highlighted in Figure 1.

For combination MBC assays, the same concentrations of antibiotics were diluted in either Schneider’s or Grace’s media, and the assays were set up as previously described for determining MIC and singular MBC. The pellets were resuspended in 500 μL instead of 100μL, and 100μL of this was plated onto each blood agar plate in triplicate for quantification. Plates were counted after 14 days of incubation at 37°C in 5% CO_2_.

### 2.4 DH82 cell culturing and antibiotic testing of intracellular *B. henselae*

DH82 cells (ATCC, CRL-10389) were thawed from frozen stocks and centrifuged at 125 × g for 6 min. Cells were washed with Eagle’s Minimum Essential Medium (EMEM) (ATCC, 30-2003) supplemented with 15% FBS (EMEM-15). Cells were then resuspended and allowed to grow for 3–5 days in T-75 flasks before passaging. DH82 cells used for *in vitro* assay testing were between passage 3 and 12.

**FIGURE 2:**
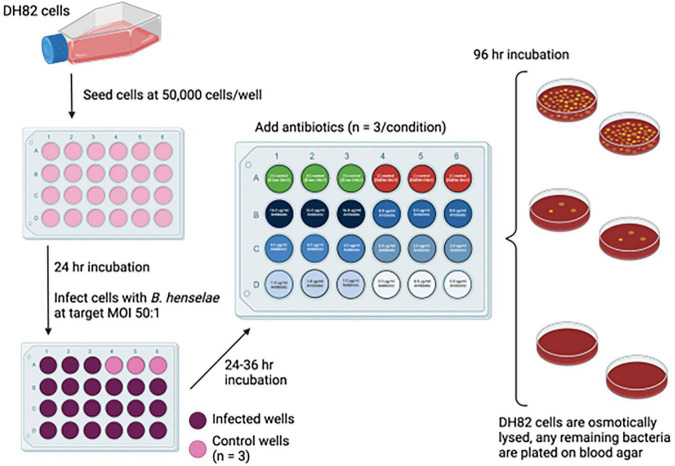
Experimental MBC assay for intracellular *B. henselae.* DH82 cells were seeded in 24-well plates at 5 × 10^4^ cells/ml. After 24 h, media was replaced with EMEM-15 containing *B. henselae* at a target MOI of 50:1 and plates were centrifuged to enhance bacteria: cell contact. Cells and bacteria were incubated for 2 h, after which remaining extracellular bacteria were removed with three PBS washes. Approximately 36–48 h later, the media in the wells was removed and replaced with media containing antibiotics at multiple concentrations. Samples were treated for 96 h, followed by washing with PBSbefore osmotic lysis using ice-cold water. The lysed contents of each well were combined and plated onto blood agar for bacterial detection. For monotherapy, the bacteria were plated on a single blood agar plate per treatment and designated as positive or negative based on growth. The bacteria were enumerated after combination antibiotic treatment by plating the undiluted DH82 lysate from each well/treatment in triplicate on blood agar plates. Wells containing only bacteria and no bacteria were used as positive and negative controls, respectively. Control wells with untreated infected cells were serially diluted before plating to obtain accurate counts. All incubations during the assay were at 37°C in 5% CO_2_. For combination treatments, statistically significant bacterial reductions were determined by comparing bacteria counts of each respective condition to the antibiotic-free control.

For assays (see Figure 2), 24 h prior to infection, cells were seeded at a concentration of 1 × 10^5^ cells/ml in a 24-well cell culture plate. On the day of infection, the OD_600_ of the *Bartonella* liquid culture was measured. The amount of culture needed to inoculate each well with an OD_600_ of 0.05 was removed and centrifuged at 6,000 × g for 10 min. The culture was then washed in 1X PBS and centrifuged again at 6,000 × g for 10 min. The pellet was resuspended in the appropriate amount of EMEM-15 for an approximate multiplicity of infection (MOI) of 1:50. The media was removed from the DH82 cells and replaced with 500 μL of EMEM-15 with an OD of 0.05 of *B. henselae*. These plates were centrifuged 1200x g for 8 min and then allowed to incubate at 37°C in 5% CO_2_ for 2 h.

After 2 h, the plates were washed 3X with 1X PBS. EMEM-15 was then added to each well, and the plates were allowed to incubate for 48–72 h at 37°C in 5% CO_2_ as previously published (Gadila and Embers, 2021). After this time, the media in the wells was replaced with EMEM-15 supplemented with the designated concentration of antibiotic(s). Wells that had no antibiotics or wells that were uninfected with *Bartonella* served as the controls. The cells were allowed to incubate for 4 days at 37°C in 5% CO_2_. The wells were then washed 3X with 1X PBS, and 100 μL (singular antibiotics) or 300 μL (combinations) of ice-cold sterile water was added to each well and set on ice for 10 min to lyse. A pipette tip was used to scrape the bottom of the well and vigorously pipette the water in the plate. The replicates were combined, and the total volume (singular antibiotics) was plated on a blood agar plate or 100 μL was plated in triplicate (combination antibiotics) for quantification. Plates were checked after 10–14 days when visible colonies were present.

### 2.5 Antibiotic testing on *Bartonella* biofilms

To set up biofilm growth, Collagen I (Corning, 354231) was diluted to 7.58 μg/cm^2^ in 0.01 M HCl. Using a culture treated 96-Well plate, 50 μL of Collagen I solution was added to each well. These plates were incubated at room temperature for 1 h, then were rinsed 2 times with sterile 1X PBS. Plates were sterilized with UV light for 15 min before use or storage at 4°C for up to 7 days.

**FIGURE 3:**
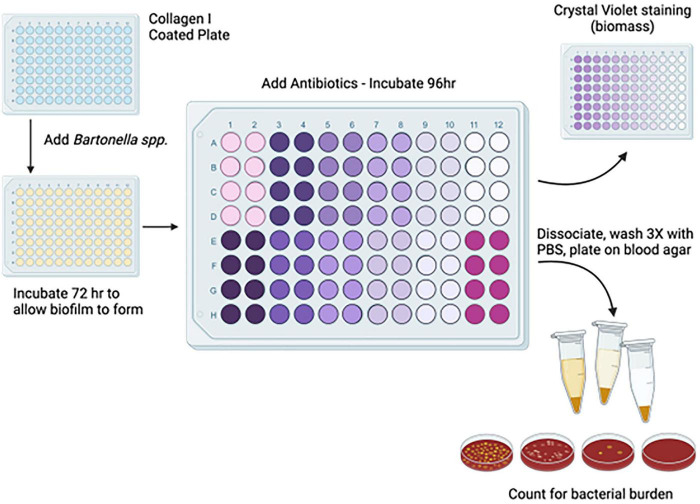
Schematic of *B. henselae* biofilm MBC assay. Biofilm matrices were generated by coating plates with Bovine Collagen I. Approximately 200 μL of Schneider’s Media with 3 × 10^5^ CFU/mL of *B. henselae* was added to the plates and allowed to form biofilms for 72 h before the media in the wells was replaced with media containing antibiotics. After 96 h of treatment, the films were washed with PBS three times and biofilms were disrupted with vigorous pipetting and scraping. The remaining bacteria were combined and plated onto blood agar plates. For monotherapy, MBC was determined by plating the entirety of the remaining bacteria on one plate and designated as positive or negative based on any bacterial growth. In combination experiments, remaining bacteria were plated in triplicate for enumeration. Significance in the MBC of antibiotic combinations was determined by comparing the number of remaining bacteria to the counts in the antibiotic-free control.

On the initial day of the assay (Figure 3), *B. henselae* was grown as described in bacterial culturing. The 0.1 OD_600_ inoculum was further diluted 1:100 for biofilms at an approximate concentration of 3 × 10^5^ CFU/ml. In each well, 200 μL of diluted bacteria was added and allowed to incubate for 3 days at 37°C in 5% CO_2_. Wells containing only Schneider’s media served as a negative control. After incubation, the supernatant in each well was aspirated, with care to not disturb the bottom or sides of the wells. The media was replaced with fresh Schneider’s medium with the appropriate dilution of antibiotics. Schneider’s media without antibiotics was added to the control wells. The plates were then incubated for an additional 4 days at 37°C in 5% CO_2._ After incubation, the plates were washed three times with 1X PBS, with care taken to not disturb the bottom or sides of the wells. After washing, 150 μL of sterile PBS was used to vigorously wash the bottom and sides of the well. The bottom and sides were also scraped with a pipette tip to ensure maximum recovery of the biofilms. The replicates were combined into a single tube and 100 μL was plated onto blood agar plates in triplicate. Plates were quantified after 10–14 days of incubation at 37°C in 5% CO_2_. Biofilm formation was optimized in preliminary experiments using crystal violet staining (Supplementary Figure 1).

## 3 Results

### 3.1 Monotherapies only inhibit growth of extracellular *Bartonella henselae*

**FIGURE 4:**
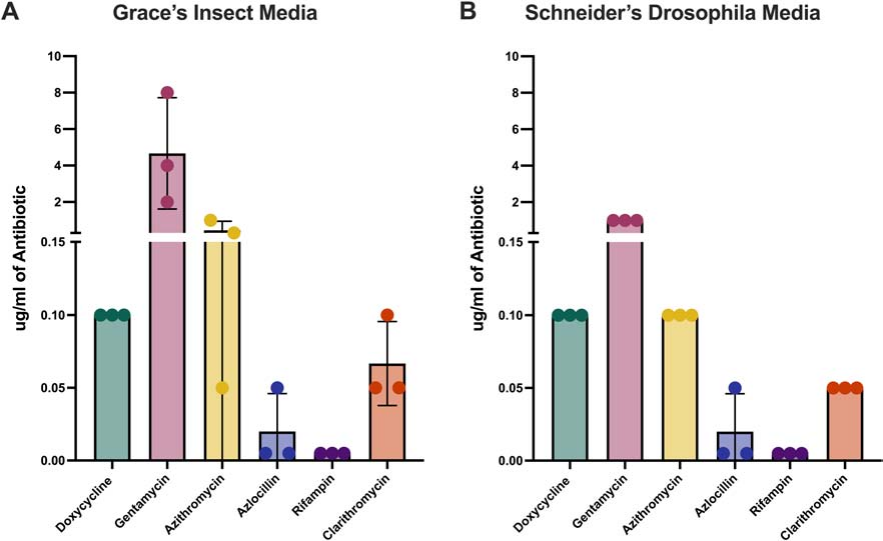
Minimum Inhibitory Concentrations for extracellular *B. henselae*. Monotherapies of doxycycline, gentamicin, azithromycin, azlocillin, rifampin, and clarithromycin were tested against extracellular *B. henselae* grown in either Grace’s **(A)** or Schneiders **(B)** Media. Concentrations of antibiotics shown on the y-axis represent the upper limit of the range in which bacterial growth was no longer detected through absorbance value determinations. These experiments were repeated three times and values for each test are represented by one symbol. The starting inoculation dose and antibiotic-free control for each assay was approximately the same for each replicate (Figures 5K, 6M).

The MICs appeared similar for doxycycline, azlocillin, and rifampin against *B. henselae* grown in either Grace’s or Schneider’s Insect Media (Figure 4 and Table 3). However, *B. henselae* had higher MICs to gentamicin, azithromycin, and clarithromycin when grown in Grace’s Insect Media compared to Schneider’s. The lowest MIC against *B. henselae* was observed in media supplemented with rifampin, followed by azlocillin. The highest MIC in both medias was gentamicin. Concentrations of antibiotics tested are reflective of the concentration of antibiotic typically achieved in patient serum (National Academies of Sciences Engineering and Medicine, 2020) with standard doses. Data points are reflective of the higher range interval which showed no growth in three separate iterations of these experiments. For example, if gentamicin-treated *B. henselae* grew at 4 μg/mL but not at 8μg/mL, then the point is set to 8 μg/mL on the graph. It is important to note that there was variation in results between each iteration of the experiment, particularly when *B. henselae* was grown in Grace’s Media (Figure 4A). This is perhaps reflective of the sensitive and variable nature in which *Bartonella* grows in different culture medias, and there is no accepted standard for growing these bacteria.

**TABLE 3:** *Bartonella henselae* minimum bactericidal concentrations.

Monotherapy efficacy against *B. henselae*						
	Minimum inhibitory concentration (μg/mL)		Minimum bactericidal concentration (μg/mL)			
Antibiotic	Grace’s media (extracellular)	Schneider’s media (extracellular)	Grace’s media (extracellular)	Schneider’s media (extracellular)	Intracellular (DH82 cells)	Biofilm
Rifampin	0.001–0.005	0.0001–0.0005	>10.0	>10.0	>16.0	> 16.0
Clarithromycin	0.01–0.1	0.01–0.05	>10.0	>10.0	>16.0	> 16.0
Doxycycline	0.01–0.1	0.01–0.1	>10.0	>10.0	>16.0	> 16.0
Gentamicin	2.0–4.0	0.5–1.0	8.0–16.0	8.0–16.0	>16.0	> 16.0
Azithromycin	0.05–0.5	0.05–0.1	>10.0	>10.0	>16.0	> 16.0
Azlocillin	0.001–0.05	0.001–0.005	>5.0	>5.0	>16.0	> 16.0

Most singular antibiotic treatments against *B. henselae* were ineffective at killing the bacteria in lower concentrations as seen in Table 3, although higher concentrations of the aminoglycoside gentamicin were effective at killing bacteria. This is surprising because rifampin and azlocillin are bactericidal as well. It would be expected that the bactericidal antibiotics killed *B. henselae* at higher concentrations while the bacteriostatic antibiotics did not. Monotherapy was also ineffective at treating *B. henselae* residing in DH82 canine macrophages and when *B. henselae* was grown as a biofilm, as is shown in Table 3. Indeed, the majority of blood agar plates from the biofilm assays contained too many colonies to count (approximately at least 300 colonies), regardless of concentration of antibiotic used (not shown).

**FIGURE 5:**
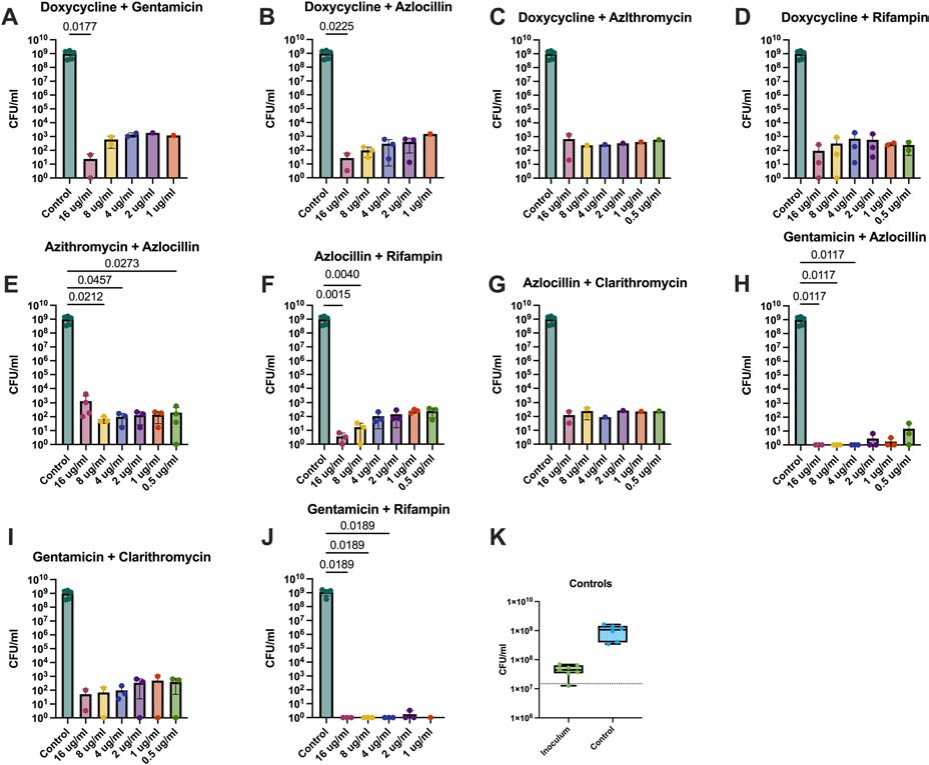
Combination therapy against extracellular *B. henselae* grown in Schneider’s Media. Dual combination therapy against extracellular *B. henselae* grown in Schneider’s Media yielded countable results in 9 different combinations at various concentrations: **(A)** Doxycycline and gentamicin, **(B)** doxycycline and azlocillin, **(C)** doxycycline and azithromycin, **(D)** doxycycline and rifampin, **(E)** azithromycin and azlocillin, **(F)** azlocillin and rifampin, **(G)** azlocillin and clarithromycin, **(H)** gentamicin and azlocillin, **(I)** gentamicin and clarithromycin, and **(J)** gentamicin and rifampin. Concentrations of antibiotics were a 1:1 ratio of the concentration listed on the x-axis. Effective bactericidal activity is shown as significant *p*-values generated from Kruskal-Wallace tests with Dunn’s corrections for multiple comparisons and show comparison between the saline control group and the respective concentration. Concentrations of 0.05 μg/mL not listed **(A,B,J)** are indicative of results that were unable to be enumerated. The inoculation dose and the saline controls are represented in **(K)**, with the dotted line on the graph representing the target inoculation dose.

**FIGURE 6:**
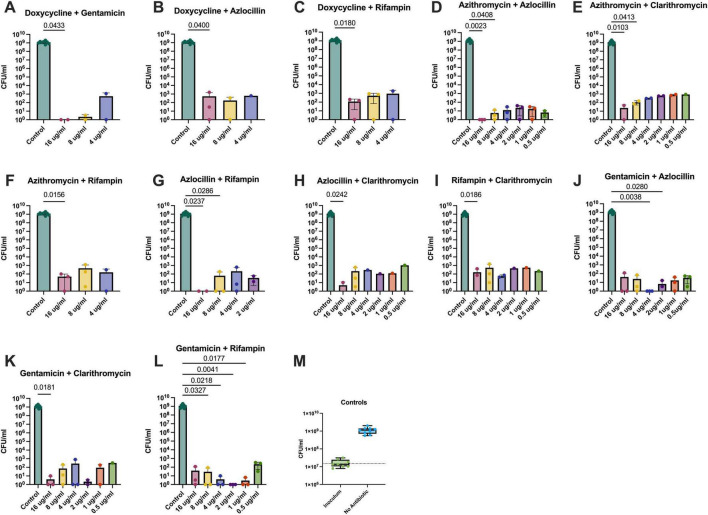
Combination therapy against extracellular *B. henselae* grown in Grace’s Media: **(A)** Doxycycline and gentamicin, **(B)** doxycycline and azlocillin, **(C)** doxycycline and rifampin, **(D)** azithromycin and azlocillin, **(E)** azithromycin and clarithromycin, **(F)** azithromycin and rifampin, **(G)** azlocillin and rifampin, **(H)** azlocillin and clarithromycin, **(I)** rifampin and clarithromycin, **(J)** gentamicin and azlocillin, **(K)** gentamicin and clarithromycin, and **(L)** gentamicin and rifampin. A total of 12 different dual therapies yielded countable reductions in *B. henselae* burden when grown extracellularly in Grace’s Media. Concentrations of antibiotics were a 1:1 ratio of the concentration listed on the x-axis. Specific combinations and concentrations are indicated by a statistically significant reduction in bacteria compared to saline controls. *P*-values shown are generated from Kruskal-Wallace tests with Dunn’s corrections for multiple comparisons and show comparison between the saline control group and the respective concentration that produced a value less than *p* = 0.05. Concentrations not listed from the x-axes in **(A–C,F,G)** indicate that those concentrations yielded agar plates which could not be enumerated. The bacterial counts for the starting dose of *B. henselae* and the saline treated group are shown in **(M)**.

Extracellular bactericidal activity was measured in both Schneider’s (Figure 5) and Grace’s (Figure 6) insect medias as both reagents are used in the literature. The most effective combinations were azithromycin and azlocillin (Figures 5E, 6D) as well as gentamicin in combination with either azlocillin (Figures 5H, 6J) or rifampin (Figures 5J, 6L), as these combinations reduced *B. henselae* loads significantly in both medias. Efficacy in this context is evidenced by significant bacterial reductions at lower concentrations of antibiotics when compared to saline controls. Gentamicin, azlocillin, and rifampin are all bactericidal antibiotics, which may explain the increased efficacy in these combinations when compared to doxycycline combinations. Azithromycin and azlocillin, the combination that our lab has previously shown to be effective against *B. henselae* (Gadila and Embers, 2021), was recapitulated in these experiments (Figures 5E, 6D), with similar results.

### 3.2 Combining antibiotics enhances their potency against *B. henselae* in its extracellular form

In general, *B. henselae* was sensitive to more antibiotic combinations when grown in Grace’s media (Figure 6) compared to Schneider’s media (Figure 5). In Grace’s media, *B. henselae* was susceptible to 12 different combinations tested, as is indicated by at least one concentration yielding significantly lower colony forming units compared to saline controls (Figure 6). In comparison, only 6 different combinations were effective at significantly reducing *B. henselae* growth in Schneider’s media (Figure 5).

It is important to note that doxycycline and gentamicin, the most commonly prescribed antibiotic combination for severe *Bartonella* infections, significantly reduced *B. henselae* bacteria at 16μg/ml only (Figures 5A, 6A). This effective level is well above the maximum serum concentration (2.6 μg/mL) achievable with the highest clinically safe dose of doxycycline (200 mg) (National Academies of Sciences Engineering and Medicine, 2020). These results suggest that current dosing regimens for this antibiotic may be inadequate for effectively eradicating severe *Bartonella* infections in patients. Furthermore, the only other doxycycline combination which effectively reduced extracellular growth was in combination with azlocillin (Figures 5B, 6B) or rifampin (Figure 6C), and again only at the highest concentration tested. Taken together, these results highlight the conflicting differences between *in vitro* evidence of doxycycline efficacy and the continuing recommendation of doxycycline as one of the primary drugs of choice against *Bartonella* infections in patient care.

The combinations which demonstrate bactericidal activity at lower concentrations are more reflective of achievable serum levels when administered to patients. These combinations, azithromycin with azlocillin and gentamicin with either azlocillin or rifampin, were selected for further testing against *B. henselae* in different microenvironments.

### 3.3 Intracellular *B. henselae* is susceptible to aminoglycosides in combination with other bactericidal antibiotics.

**FIGURE 7:**
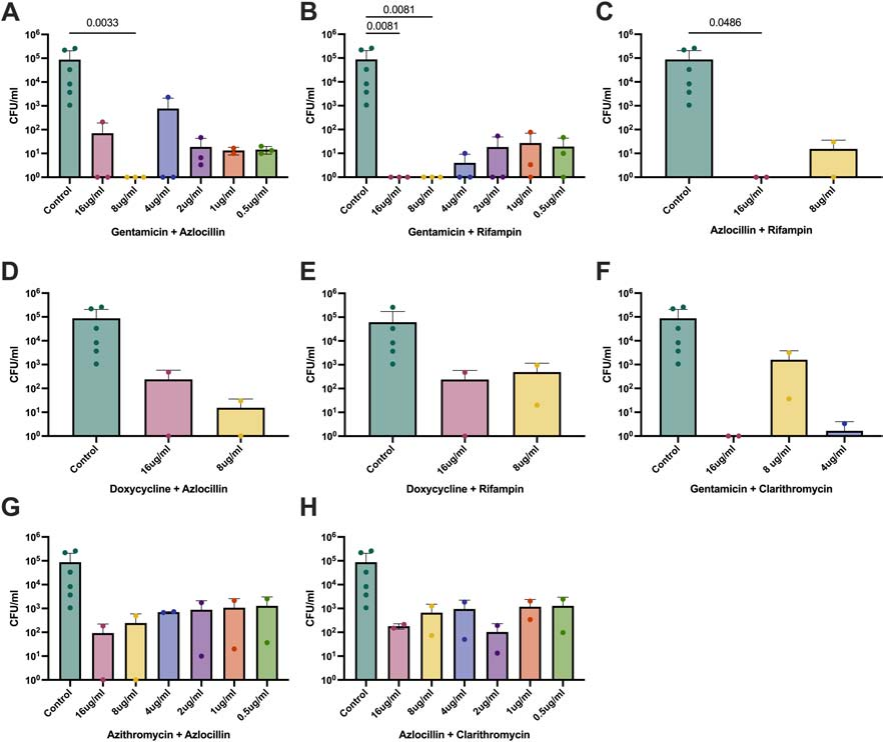
Bactericidal activity of dual antibiotic therapy in *B. henselae*-infected DH82 cells. Antibiotic combinations that were effective against extracellular *B. henselae* in both medias were examined for bacterial killing of *B. henselae* inside DH82 canine macrophages. Of the 8 combinations used, only gentamicin in combination with azlocillin **(A)** or rifampin **(B)** or azlocillin and rifampin **(C)** were able to significantly reduce intracellular *B. henselae* at any concentration tested. The other following combinations are shown: **(D)** doxycycline and azlocillin, **(E)** doxycycline and rifampin, **(F)** gentamicin and clarithromycin, **(G)** azithromycin and azlocillin, and **(H)** azlocillin and clarithromycin. Lower concentrations of antibiotics yielded plates with bacteria that were too numerous to count (TNTC) for many combinations, so these bars have been excluded from the figure. Successful bacterial reduction was determined as a statistically significant reduction in bacterial counts of that particular condition compared to the saline controls. *P*-values were generated using the Kruskall-Wallace test with Dunn’s correction for multiple comparisons.

Gentamicin in combination with either azlocillin (Figure 7A) or rifampin (Figure 7B) was effective in treating intracellular infection (Figure 7), as measured by significant reductions in bacterial counts. This is unusual, but expected, as gentamicin (Chomel et al., 2003), azlocillin (Liao et al., 2019), and rifampin (Patel and Alan, 2023) have all been used to treat intracellular infections, despite evidence demonstrating poor uptake into host cells. Treatment with azlocillin and rifampin (Figure 7C) was also shown to be significant at 16 μg/mL, but concentrations lower than 8 μg/mL did not reduce bacterial burden and were unable to be counted and graphed. Azlocillin with azithromycin, which was shown to be effective against extracellular *B. henselae* at multiple concentrations and in previous work (Gadila and Embers, 2021), was only moderately effective against intracellular bacterial infection (Figure 7G). Therefore, only gentamicin with rifampin and gentamicin with azlocillin were used in further testing against *B. henselae* biofilms.

### 3.4 *B. henselae* in biofilm requires higher doses of effective antibiotic combinations to stop growth

**FIGURE 8:**
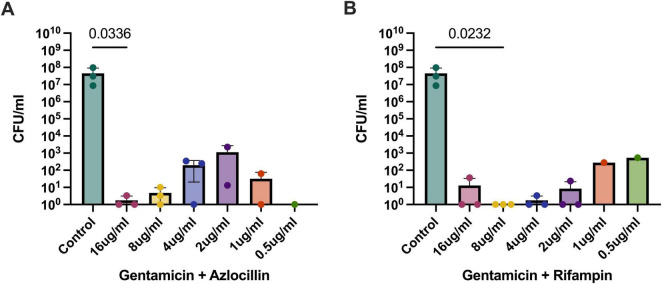
Combination treatment for *B. henselae* biofilms. Gentamicin in combination with azlocillin **(A)** or rifampin **(B)** significantly reduced *B. henselae* biofilm counts at higher concentrations. *P*-values shown were generated using the Kruskall-Wallace test with Dunn’s correction for multiple comparisons and compare that specific concentration to the saline control.

Both combinations of antibiotics were efficacious at reducing *B. henselae* biofilms, albeit only at 16 μg/mL (gentamicin and azlocillin, Figure 8A) and 8 μg/mL (gentamicin and rifampin, Figure 8B). All of the antibiotics tested in these experiments are bactericidal, so elimination of *B. henselae* biofilms shows promise for effective treatment in future animal studies. An interesting trend showing susceptibility of *B. henselae* to the drug combinations at the higher and lower doses indicates that there is significant variability; more replicates may be needed, and the optimal doses may be significantly different between to the two drugs which were combined.

## 4 Discussion

In these experiments, the efficacy of monotherapy and combination antibiotic regimens was evaluated against extracellular *Bartonella henselae*. For monotherapy, gentamicin was the only antibiotic for which a reportable Minimum Bactericidal Concentration (MBC) fell within the testing range, despite its Minimum Inhibitory Concentration (MIC) being the highest among the tested drugs. This finding may be attributable to the bactericidal and concentration-dependent nature of this drug. Furthermore, these findings demonstrate the critical importance of testing for bactericidal activity in *Bartonella* species and not relying solely on MIC data to determine antibiotic efficacy. We did not perform experiments to establish the MIC values of intracellular bacteria or biofilms. One previous study demonstrated that clarithromycin was bacteriostatic against *B. henselae* and *B. quintana* inside Vero cells (Ives et al., 2000), however we did not see bactericidal activity inside our DH82 model. It will be important to perform these assays in other cell lines to obtain a better picture of how *Bartonella* species behaves relative to other cell types. Correlation of efficacy *in vitro* to *in vivo* is difficult to gauge and is even further complicated by differences between clinical studies that show various achievable serum levels, use different antibiotic doses, and utilize different administration routes.

For combination therapy, gentamicin combined with azlocillin or rifampin appeared highly effective against extracellular *B. henselae*. This finding is supported by the known synergy between beta-lactams (like azlocillin) and aminoglycosides (like gentamicin) against gram-negative bacteria, further rationalizing their combined use for *Bartonella* infections. This is an interesting finding since it is well-established that gentamicin is unable to cross eukaryotic cell membranes. Another study examining the efficacy of different antibiotics against *B. quintana* inside erythrocytes found similar results with bactericidal activity of gentamicin against intracellular bacteria (Rolain et al., 2003). Importantly, the most commonly prescribed antibiotic combination in the clinic, doxycycline plus gentamicin, was not effective against intracellular or biofilm-grown *B. henselae*. In this manuscript, we defined “successful” bactericidal activity as a statistically significant reduction in bacteria counts instead of the 99.9% standard. While many antibiotics are found effective against *Bartonella in vitro*, numerous case reports exemplify that this is not the case *in vivo*. It is our hope that using these rigorous measurements to define efficacy may better correlate to successful elimination of Bartonella bacteria in future animal studies.

While azlocillin and azithromycin were effective against extracellular *B. henselae*, their efficacy was significantly reduced when the bacteria were grown inside DH82 cells. Like many other intracellular pathogenic bacteria, *B. henselae* effectively resides inside host cells, avoidingcontact with antibiotics. *In vitro* experiments have shown that *B. henselae* resides in endosomes within endothelial and macrophage cell lines, similar to the evasion mechanism used by *Mycobacteria* (Carryn et al., 2003; Kyme et al., 2005), which are also commonly treated with rifampin. Biofilms are a major cause of antibiotic resistance, primarily due to the physical blockade that limits antimicrobial agents access to the bacterial cells. *B. henselae* is capable of forming biofilms in both cat flea guts and mammalian hosts via the BadA autotransporter gene (Okaro et al., 2017; Okaro et al., 2019; Okaro et al., 2021). In fact, this virulence factor is implicated in numerous pathogenesis strategies for *B. henselae* within the host, mostly involving adhesion and invasion into cells (Xi et al., 2024). Other *in vitro* studies have found that rifampin and azithromycin combinations were effective at completely eliminating *B. henselae* biofilms, but only after treatment of 6 full days (Zheng et al., 2020).

## 5 Limitations and future directions

The dual therapy antibiotic concentrations were only examined for bactericidal effects in a 1:1 ratio. Future experiments should focus on refining dual or even triple therapy combinations to identify a treatment regimen using the lowest possible concentrations. These experiments could include checkerboard synergy assays to assess fractional inhibitory concentrations (FIC). Additionally, we did not perform MIC assays to specifically assess *Bartonella* growth inhibition in either biofilm or intracellular environments. A more refined regimen could potentially reduce the risk of side effects and drug toxicity.

One limitation of the experiments described in this paper is that these experiments were performed without serial dilutions to quantify greater counts of live bacteria. This has led to some conclusions being qualitatively described as “too numerous to count” in lieu of providing counts with statistical conclusions. This limits our knowledge of the functionality of these antibiotics, as it is possible the bacteria load could have been lowered 100-fold or possibly even 1,000-fold. However, a reduction in bacterial load may or may not be enough for effective treatment in human patients; thus, the overall conclusion would not change with these counts. Many patients with serious *Bartonella* infections have suppressed immune systems, so clearance of bacteria is more imperative than a reduction in bacterial load.

*Bartonella* species have been shown to infect multiple cell types, including erythrocytes (Schulein and Dehio, 2002) and endothelial cells (Xi et al., 2024) in addition to macrophages such as the canine DH82 cells used in these experiments. Thus, testing antibiotic efficacy in a canine macrophage-like cell line is not fully representative of the microenvironment of intracellular *Bartonella* infection, particularly in human hosts. It will be important to test antibiotic efficacy against *Bartonella* inside cells that do not perform phagocytosis, as *Bartonella* uses different mechanisms to enter these cells and antibiotic uptake into host cells would also likely be different. Subsequent *in vivo* studies must also take the human equivalent dose and pharmacokinetics into account. Most of these drugs have not been established pharmacokinetically in models such as immune-deficient mice, dogs and cats. The overarching goal will be for the efficacy of these antibiotic combinations to be tested in relevant animal models and eventually in human trials, if warranted.

## Data Availability

The original contributions presented in this study are included in this article/Supplementary material, further inquiries can be directed to the corresponding author.

## Supplementary material

Supplemental Figure 1 Optimizing *B. henselae* biofilm growth using crystal violet staining and calculated biomass. Biofilm growth of *B. henselae* used these experiments was optimized by measuring crystal violet mass and bacterial colony counts on multiple days. Using similar conditions tested in Okaro (Okaro et al., 2019) and from collaborators (not published), we decided to test *B. henselae* biofilm formation after 3, 5, 7, and 14 days with wells coated in collagen I (Corning, 354231). Biofilm growth was measured by relative biomass compared to uninoculated but treated wells as controls. Plates were stained with crystal violet solution as previously established (O’Toole, 2011), then washed with acetone before the absorbance at OD_550_ was read. The difference between inoculated and control absorbances are shown for both Schneider’s Media **(A)** and Grace’s Media **(C)**. Additionally, biomass of *B. henselae* was calculated using bacterial counts and total area of each well in either Schneider’s Media **(B)** or Grace’s Media **(D)**. From this experiment we determined that 72 hours of growth in collagen I coated plates is sufficient for the formation of a biofilm, and that peak CFU counts would occur at the end of treatment period of 96 hours (a total of 7 days post inoculation) in Schneider’s Media.
