# Supplementary figures and images for: Combination antibiotic therapy is required to eliminate Bartonella henselae in multiple microenvironments
Source: Front Microbiol. 2026 Apr 2;17:1726180. doi: 10.3389/fmicb.2026.1726180 (PMC13083074; doi:10.3389/fmicb.2026.1726180)

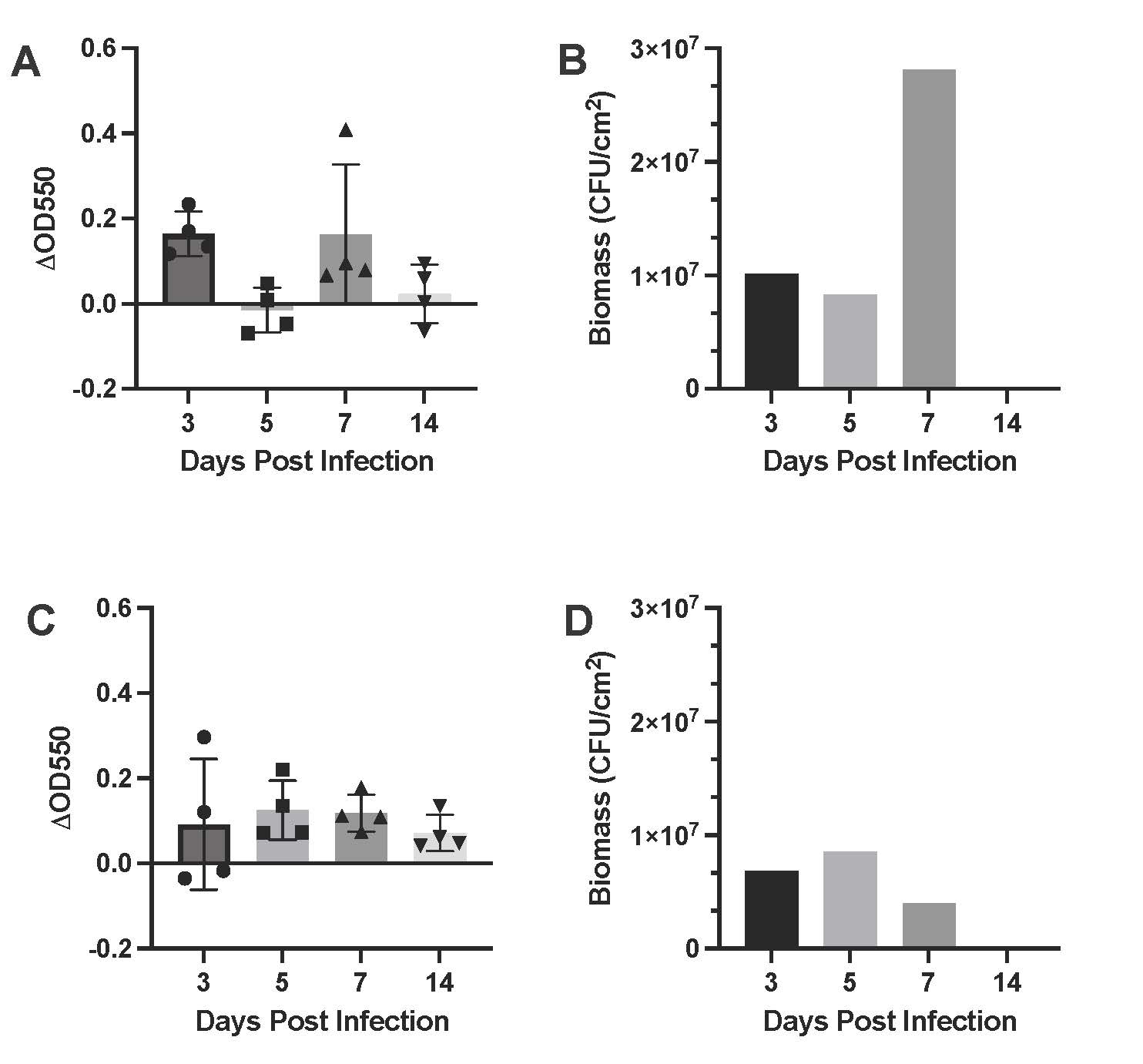

Supplement: Supplemental Figure 1 — Optimizing B. henselae biofilm growth using crystal violet staining and calculated biomass. Biofilm growth of B. henselae used these experiments was optimized by measuring crystal violet mass and bacterial colony counts on multiple days. Using similar conditions tested in Okaro (Okaro et al., 2019) and from collaborators (not published), we decided to test B. henselae biofilm formation after 3, 5, 7, and 14 days with wells coated in collagen I (Corning, 354231). Biofilm growth was measured by relative biomass compared to uninoculated but treated wells as controls. Plates were stained with crystal violet solution as previously established (O’Toole, 2011), then washed with acetone before the absorbance at OD550 was read. The difference between inoculated and control absorbances are shown for both Schneider’s Media (A) and Grace’s Media (C). Additionally, biomass of B. henselae was calculated using bacterial counts and total area of each well in either Schneider’s Media (B) or Grace’s Media (D). From this experiment we determined that 72 hours of growth in collagen I coated plates is sufficient for the formation of a biofilm, and that peak CFU counts would occur at the end of treatment period of 96 hours (a total of 7 days post inoculation) in Schneider’s Media. [file Image_1.JPEG]
